# Quality of life in patients with intermittent claudication

**DOI:** 10.1007/s00772-017-0269-4

**Published:** 2017-04-18

**Authors:** A. E. Harwood, J. P. Totty, E. Broadbent, G. E Smith, I. C. Chetter

**Affiliations:** Academic Vascular Surgical Unit, Hull Royal Infirmary, Anlaby Road, HU3 2JZ Hull, UK

**Keywords:** Ankle brachial index, Exercise, Peripheral arterial disease, Questionnaire, Review, Knöchel-Arm-Index, Bewegung, Periphere arterielle Verschlusskrankheit, Fragebogen, Review

## Abstract

**Background:**

Intermittent claudication (IC) is a common condition that causes pain in the lower limbs when walking and has been shown to severely impact the quality of life (QoL) of patients. The QoL is therefore often regarded as an important measure in clinical trials investigating intermittent claudication. To date, no consensus exits on the type of life questionnaire to be used. This review aims to examine the QoL questionnaires used in trials investigating peripheral arterial disease (PAD).

**Material and methods:**

A systematic review of randomised clinical trials including a primary analysis of QoL via questionnaire was performed. Trials involving patients with diagnosed PAD were included (either clinically or by questionnaire). Any trial which had QoL as the primary outcome data was included with no limit being placed on the type of questionnaire used.

**Results:**

The search yielded a total of 1845 articles of which 31 were deemed appropriate for inclusion in the review. In total, 14 different QoL questionnaires were used across 31 studies. Of the questionnaires 24.06% were missing at least one domain when reported in the results of the study. Mean standard deviation varied widely based on the domain reported, particularly within the SF36.

**Discussion:**

Despite previous recommendations for Europewide standardisation of quality of life assessment, to date no such tool exists. This review demonstrated that a number of different questionnaires remain in use, that their completion is often inadequate and that further evidence-based guidelines on QoL assessment are required to guide future research.

## Zusammenfassung

**Hintergrund:**

Die Claudicatio intermittens (CI) ist eine häufige Erkrankung, die beim Gehen Schmerzen in der unteren Extremität verursacht und die Lebensqualität (QoL) der Patienten nachweislich beeinträchtigt. Daher wird die QoL oft als wichtiges Messinstrument in klinischen Studien angesehen, welche die Claudicatio intermittens untersuchen. Bis heute gibt es keinen Konsens bezüglich der Art des zu verwendenden Lebensqualitätsfragebogens. Ziel dieses Reviews ist es, die Fragebögen zur Lebensqualität zu untersuchen, die in Studien zur peripheren arteriellen Verschlusskrankheit (PAVK) zum Einsatz kommen.

**Material und Methoden:**

Ein systematischer Review von randomisierten klinischen Studien einschließlich einer QoL-Primäranalyse mittels Fragebogen wurde durchgeführt. Studien an Patienten mit diagnostizierter PAVK wurden eingeschlossen (entweder klinisch oder mittels Fragebogen). Jede Studie, die QoL als primären Endpunkt hatte, wurde eingeschlossen, ohne Limitierung hinsichtlich der Art des verwendeten Fragebogens.

**Ergebnisse:**

Die Suche ergab insgesamt 1845 Artikel, von denen 31 für den Einschluss in die Studie als geeignet befunden wurden. Insgesamt 14 verschiedene QoL-Fragebögen kamen in den 31 Studien zum Einsatz. Bei 24,06% der Fragebögen fehlte mindestens eine Domäne in den berichteten Studienergebnissen. Die mittlere Standardabweichung fiel, je nach berichteter Domäne, sehr unterschiedlich aus, insbesondere im SF36.

**Diskussion:**

Trotz früherer Empfehlungen für eine europaweite Standardisierung der Beurteilung der Lebensqualität, existiert hierfür bis heute kein Instrument. Dieser Review zeigte, dass weiterhin etliche verschiedene Fragebögen verwendet werden, dass deren Vollständigkeit häufig inadäquat ist und dass weitere evidenzbasierte Leitlinien zur Beurteilung der Lebensqualität notwendig sind, um für die zukünftige Forschung wegweisend zu sein.

## Introduction

Peripheral arterial disease (PAD) is a common chronic condition that can cause lower extremity pain when walking; classically known as intermittent claudication (IC). Clinically patients have diminished or absent pulse on physical examination and an ankle-brachial pressure index (ABPI) of <0.9 [28]. The prevalence of PAD is around 4% increasing with age, gender, ethnicity and rises up to and above 10% over the age of 70 years [27].

The impact of PAD on quality of life has been well demonstrated [31], with IC not only affecting walking distance, capacity and physical activity but social function, emotional well-being and mental health [35]. The primary treatment aim is therefore not only to improve blood flow into the leg but also the quality of life for the patient. National governing bodies recommend a supervised exercise programme as the first line treatment, along with best medical therapy [25]. If supervised exercise is not feasible, acceptable or accessible for patients [17] then more invasive therapies, such as angioplasty or bypass surgery may be utilised. Quality of life (QoL) is an important outcome measure with the World Health Organization (WHO) defining it as “physical, social and mental well-being and not just an absence of infirmity” [31]. Since QoL is an important outcome indicator of treatment success, most clinical trials include some form of QoL measure amongst their outcomes. The QoL can be measured with either generic or disease-specific questionnaires and although there are a multitude of questionnaires available for use in the PAD population, no consensus exists as to which questionnaire is the most appropriate in this group. To date, there has been no systematic review of QoL assessment methods and outcomes in clinical trials involving claudicants or following interventional procedures for PAD. The following review aims to correct this deficit in the literature.

## Methods

### Search strategy

A systematic review of randomised clinical trials including a primary analysis of QoL via questionnaire was performed. The Preferred Reporting Items for Systematic Reviews and Meta-Analyses (PRISMA) guidelines was used for reporting search results.

### Inclusion criteria

Trials involving patients with diagnosed PAD were included (either clinically or by questionnaire). Any study involving patients with critical limb ischemia or self-reported PAD status was excluded. Any trial which had QoL as the primary outcome data was included with no limit being placed on the type of questionnaire used.

### Database search

This systematic search of the MEDLINE, CENTRAL and Embase databases was performed. The search strategy aimed to include any trial where QoL was specified as the primary outcome measure. Search terms used were: “intermittent claudication” [OR] “peripheral arterial disease” [AND] “QUALITY OF LIFE” [OR] “SF36” [OR] “QUESTIONNAIRE” [OR] “EQ5D” [OR] “VASCUQOL”.

Searches were limited to run from 1947 to September 2016, full text articles related to adults over the age of 18 years and published in the English language. Abstracts were independently assessed for relevance by two reviewers (A. H & J. T). Citations from the full texts of relevant reports were hand searched for other relevant references.

### Data extraction

Data were extracted from full text articles by two investigators (A. H & J. T) using a standardised data extraction excel spreadsheet. Any disagreement as to inclusion of an article between the two assessing investigators was settled by consensus with a third investigator (G. S).

## Results

### Search results

**Fig. 1:**
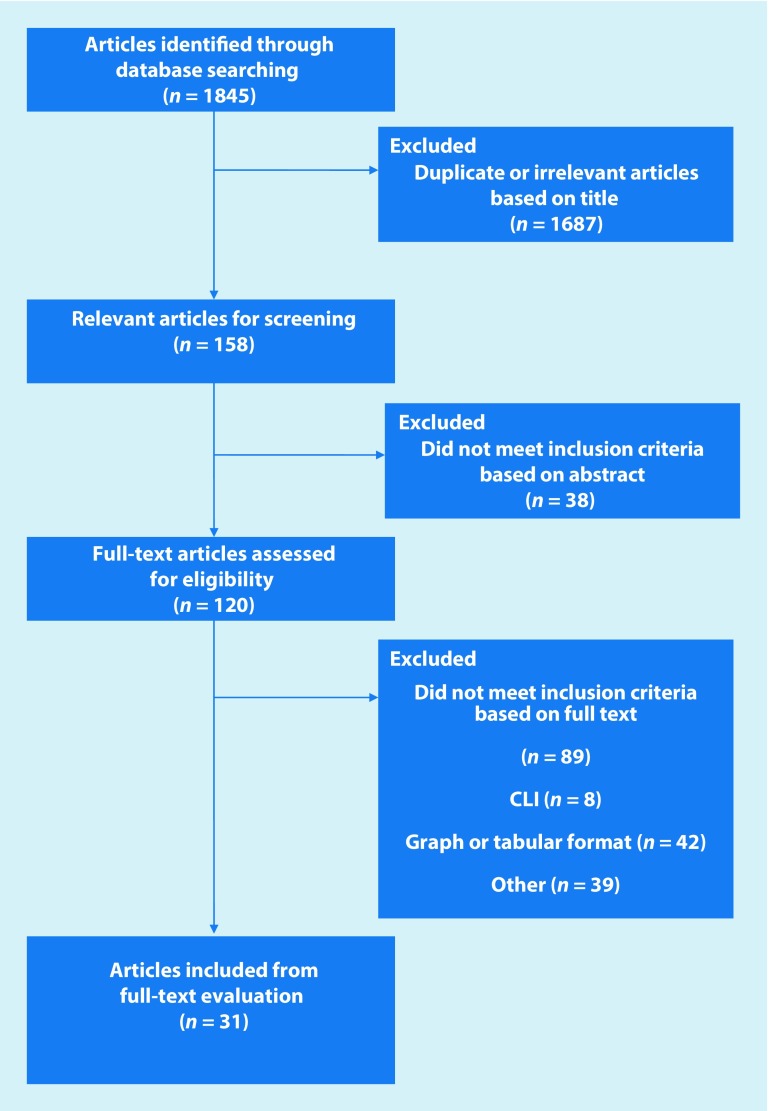
Prisma flow diagram of literature search process. *CLI* critical limb ischemia

As summarised in Fig. 1, the search yielded a total of 1845 articles of which 31 were deemed appropriate for inclusion in the review (Table 1).

**Table 1:** Summary of the study characteristics including all values for PAD patients (ABPI is taken at rest in the worst affected limb an data are presented as mean ±SD)

	Author, year	Sample size (*n*)	Age (years)	Males (%)	ABPI	Intervention	QoL used
1	Shuriquie et al. [37]	96	59 ± 15.6	65 (67.7)	Not reported	Bypass surgery	AUSVIQOL – Arabic
2	Haitjema et al. [16]	865	68	623 (72.0)	0.59 ± 0.21	Endarterectomy	SF36
3	Prévost et al. [36]	46	60.3 ± 8	40 (87.0)	0.7 ± 0.1	Home exercise programme	SF36 – French
4	Maksimovic et al. [28]	102	68.9 ± 8	33 (32.4)	Not reported	None	SF36 – Serbian
5	Inglis et al. [21]	173	60.5 ± 15.4	70 (40.5)	Not reported	None	SF12
6	Guidon and McGee 2013 [14]	44	67 ± 8	33 (75.0)	0.77 ± 0.21	Supervised exercise	WIQ SF36 ICQ
7	Fritschi et al. [12]	105	68.9 ± 8.35	64 (61.0)	Not reported	None	SF36 WIQ
8	Frans et al. [11]	40	67	25 (62.5)	0.67 ± 0.21	None	SF36 VascuQoL ALDS
9	Lee et al. [26]	63	55 ± 12	15 (23.8)	Not reported	None	PAQ
10	Fakhry et al. [10]	217	67.5 ± 9.5	135 (62.2)	0.62 ± 0.19	Home exercise programme vs. supervised exercise programme	SF36 EuroQoL VascuQoL
11	Malagoni et al. [29]	250	70.5 ± 9.2	191 (76.4)	Not reported	Home exercise programme	SF36
12	Yan et al. [42]	134	71 ± 9	94 (70.1)	0.6 ± 0.2	None	WIQ
13	Hedeager Momsen et al. [19]	88	67.4 ± 6.9	56 (63.6)	0.53	Revascularisation	SF36 WIQ
14	Leicht et al. [27]	28	69 ± 7.6	22 (78.6)	0.7 ± 0.1	None	ICQ SF36
15	Tsai et al. [39]	53	76.2 ± 3.7	44 (83.0)	0.7 ± 0.1	Supervised exercise programme	SF36 – Chinese
16	Breek et al. [6]	151	63	100 (66.2)	Not reported	None	WHOQoL-100
17	Bosch and Hunink [4]	254	58	183 (72.0)	Not reported	None	RAND-36 HUI3 EQ5D
18	Hicken et al. [20]	96	68	64 (66.7)	>0.9	None	EQ5D
19	Mangiafico et al. [30]	42	64 ± 8	37 (88.1)	0.55 ± 0.22	Drug intervention (prostaglandin)	WIQ RAND-36
20	Bartman et al. [2]	44	70.5 ± 6	43 (97.7)	<0.9	None	SF36 HUI3 Rating scale
21	Cook and Galland [9]	24	66	12 (50.0)	Not reported	Revascularisation	EuroQoL Walking distance score Visual analog scale
22	Je et al. [22]	149	70.3 ± 9.7	125 (83.9)	0.75 ± 0.24	Revascularisation	PAQ
23	Oka and Sanders [34]	74	72 ± 7	56 (75.7)	0.67 ± 0.14	None	SF36
24	Gardner et al. [13]	201	67 ± 9	159 (79.1)	0.7 ± 0.22	None	SF36 WIQ
25	Kalbaugh et al. [23]	54	64.5 ± 11.2	Not reported	Not reported	Revascularisation	SF36
26	Nicolai et al. [33]	91	66.2 ± 9.6	56 (61.5)	0.72 ± 0.17	Supervised exercise programme	WIQ RAND-36 EuroQoL
27	Keeling et al. [24]	40	63	25 (62.5)	Not reported	Revascularisation	SF36
28	Virkkunen et al. [40]	27	69.3 ± 10.7	Not reported	0.63	Revascularisation	NHP
29	Aquarius et al. [1]	188	64.7 ± 9.9	119 (63.3)	0.61	None	RAND-36 WHOQoL-100
30	Breek et al. [5]	200	63	135 (67.5)	0.62	None	RAND-36 WHOQoL-100
31	Spertus et al. [38]	44	68 ± 11	24 (54.5)	Not reported	None	PAQ WIQ SF36

*ABPI* ankle-brachial pressure index

A range of interventions (including revascularisation, drug intervention and exercise therapy) were used in included papers alongside a variety of QoL data. The QoL data collection varied widely in both the timing of collection and the tool or questionnaire utilised. Study characteristics including sample size, age and ankle-brachial pressure indices (ABPI) and QoL data collection tools utilised are summarised in Table 1.

## Number of questionnaires used

A wide variety of QoL measuring tools were used in the studies included in the review. The most commonly used tool was the Short Form 36 (SF-36) or variations of it, used in 23 out of the 31 studies included (74.19%) with a total of 3256 patients and 12 studies used the SF-36 in its English form [2, 10–14, 19, 20, 23, 24, 34, 38]. Translated versions of the SF-36 were used in Serbian [28], Dutch [16], French [36], Italian [29] and Chinese [39]. Of the 23 studies 5 utilised the RAND-36 tool [1, 4, 5, 30, 33], which contains the same question set as the SF-36 but is analysed differently [18]. The second most common questionnaire used was the Walking Impairment Questionnaire (WIQ) used in 8 out of 31 studies (total 749 patients) [12–14, 19, 30, 33, 38, 42], including a direct comparison by Nicolai et al. of the WIQ and the SF-36. This is followed by the EuroQol questionnaire, or EQ–5D, used in 4 studies (586 patients) [4, 9, 10, 33].

In each of three studies two questionnaires were used; the Peripheral Artery Disease Quality of Life (PADQOL) tool [22, 26, 38] and the World Health Organization Quality of Life (WHOQoL-100) [1, 5, 6]. Other studies used included the Australian Vascular Quality of Life Index (AUSVIQOL) [37], the Vascular Quality of Life questionnaire (VASCUQOL) [10, 11], the Intermittent Claudication Questionnaire (ICQ) [14, 27], the Nottingham Health Profile (NHP) [40], the Health Utilities Index (HUI) questionnaire [2, 4] and Visual Analog Scales [9]. In total, 14 different QoL assessment tools were used across the 31 studies, with a total of 3928 patients surveyed.

## Number of incomplete domains

A number of studies did not fully report QoL assessment data, with several omitting domains in their final publication. Of the 23 studies using SF-36 or a variant, 6 (26.09%) reported the results of 10 domains [11, 12, 14, 23, 34, 38], including a Physical Component Summary (PCS) and Mental Component Summary (MCS). The median reported domain in the SF-36 group was 8 (range 2–10). Apart from the PCS and MCS, the most commonly omitted domain of the SF-36 was Mental Health [10, 30]. Only 1 out of 8 (12.5%) [42] studies utilising the WIQ reported pain as an outcome. Stability [30] and Activity [13] were reported in 1 out of 8 WIQ studies each, with the remaining 5 studies reporting on only 3 domains of the WIQ (62.5%) and 1 out of 3 studies using PADQOL did not report all domains, Spertus et al. [38], omitting physical function. Only 1 out of 3 studies utilising the WHOQoL-100 reported all domains, with Breek et al. [5] omitting a single domain and Aquarius et al. [1] omitting 8. Of the 31 studies in this review, 24.06% of all questionnaires were missing at least 1 domain when reported in the results of the study.

## Variance of individual results

A common theme of the extracted QoL data is individual variance of the results. A large majority (86.79%) of results were reported as mean ± standard deviation. Within each QoL questionnaire some domains have very large standard deviations, suggesting the spread of individual results is wide and therefore less reliable. In the two papers using ICQ (measured on a 0–100 scale [8]), mean standard deviation was 17.57. In studies using SF-36 or RAND-36 (that also have a total available score of 100 [41]), mean standard deviation ranged from 14.67 in the Mental Health domain to 29.41 in Role Limitation (Emotional), suggesting that some domains are more reliably interpreted than others.

## Discussion

### Number of questionnaires used and quality of study completion

Despite a recommendation for Europe-wide standardisation of QoL assessment in 1997 [7] and again in 2009 [15], this review found that a wide variety of assessment tools remain in use. These tools differ in the domains that they measure, and although there is crossover of domains in some tools [3, 4, 32], the use of lesser known QoL utilities may lead to difficulty in interpretation of any findings and comparisons between interventions.

This review showed that in those trials where a multidomain QoL utility was used (such as the SF-36, WIQ or WHOQoL-100), it was common for domains to be omitted in the final report, often without explanation. We also found that a number of different QoL assessment tools are in use in patients with PAD, and that these are often incompletely reported. Further, up to date research is needed to identify the most appropriate standard for QoL measurement that is both thorough and related to clinical outcome and acceptable for patients in terms of their ability to understand the questionnaire and the time taken to complete it.

## Limitations

This review focussed on articles where data was presented numerically for individual QoL assessment tools, and therefore could be extracted from the study for further analysis. This accounts somewhat for the high number of exclusions between full text screening (120), and the final number of papers (31) included in the review (full reasons for exclusion are shown in Fig. 1). A total of 13 studies presented their data in graphical format only, or had no data present to extract, 15 studies presented only data comparing two different QoL utilities, the majority of these presenting correlation coefficients and 9 articles did not present results as mean values, instead presenting median, mode, range or quintiles.

## Practical conclusion

Whilst QoL is regarded as an important clinical outcome measure for use in patients with PAD, this review found that standardisation of reporting QoL outcomes was poor, suggesting that a consensus on reporting standards relating to QoL measures is needed in order to guide future study design and allow more accurate comparisons between interventions.

## References

[1] Aquarius AE, De Vries J, Henegouwen DP (2006). Clinical indicators and psychosocial aspects in peripheral arterial disease. Arch Surg.

[2] Bartman BA, Rosen MJ, Bradham DD (1998). Relationship between health status and utility measures in older claudicants. Qual Life Res.

[3] Bosch JL, Halpern EF, Gazelle GS (2002). Comparison of preference-based utilities of the short-form 36 health survey and health utilities index before and after treatment of patients with intermittent Claudication. Med Decis Making.

[4] Bosch JL, Hunink MGM (2000). Comparison of the Health Utilities Index Mark 3 (HUI3) and the EuroQol EQ-5D in patients treated for intermittent claudication. Qual Life Res.

[5] Breek JC, De Vries J, Van Heck GL (2005). Assessment of disease impact in patients with intermittent claudication: discrepancy between health status and quality of life. J. Vasc. Surg..

[6] Breek JC, Hamming JF, De Vries J (2001). Quality of life in patients with intermittent claudication using the World Health Organisation (WHO) questionnaire. Eur J Vasc Endovasc Surg.

[7] Chetter IC, Spark JI, Dolan P (1997). Quality of life analysis in patients with lower limb ischaemia: suggestions for European standardisation. Eur J Vasc Endovasc Surg.

[8] Chong PF, Garratt AM, Golledge J (2002). The intermittent claudication questionnaire: a patient-assessed condition-specific health outcome measure. J Vasc Surg.

[9] Cook TA, Galland RB (1997). Quality of life changes after angioplasty for claudication: medium-term results affected by comorbid conditions. Cardiovasc Surg.

[10] Fakhry F, Spronk S, De Ridder M (2011). Long-term effects of structured home-based exercise program on functional capacity and quality of life in patients with intermittent claudication. Arch Phys Med Rehabil.

[11] Frans FA, Van Wijngaarden SE, Met R (2012). Validation of the Dutch version of the VascuQol questionnaire and the Amsterdam Linear Disability Score in patients with intermittent claudication. Qual Life Res.

[12] Fritschi C, Collins EG, O’connell S (2013). The effects of smoking status on walking ability and health-related quality-of-life in patients with peripheral arterial disease. J Cardiovasc Nurs.

[13] Gardner AW, Montgomery PS, Parker DE (2006). Metabolic syndrome impairs physical function, health-related quality of life, and peripheral circulation in patients with intermittent claudication. J Vasc Surg.

[14] Guidon M, Mcgee H (2013). One-year effect of a supervised exercise programme on functional capacity and quality of life in peripheral arterial disease. Disabil Rehabil.

[15] Gulati S, Coughlin PA, Hatfield J (2009). Quality of life in patients with lower limb ischemia; revised suggestions for analysis. J Vasc Surg.

[16] Haitjema S, De Borst G-J, De Vries J-P (2014). Health-related quality of life is poor but does not vary with cardiovascular disease burden among patients operated for severe atherosclerotic disease. IJC Heart Vessel.

[17] Harwood AE, Smith GE, Cayton T (2016). A systematic review of the uptake and adherence rates to supervised exercise programs in patients with intermittent claudication. Ann Vasc Surg.

[18] Hays RD, Sherbourne CD, Mazel RM (1993). The RAND 36-Item Health Survey 1.0. Health Econ.

[19] Hedeager Momsen AM, Bach Jensen M, Norager CB (2011). Quality of life and functional status after revascularization or conservative treatment in patients with intermittent claudication. Vasc Endovascular Surg.

[20] Hicken GJ, Lossing AG, Ameli FM (2000). Assessment of generic health-related quality of life in patients with intermittent claudication. Eur J Vasc Endovasc Surg.

[21] Inglis SC et al (2013) Angina and intermittent claudication in 7403 participants of the 2003 Scottish Health Survey: impact on general and mental health, quality of life and five-year mortality. Int J Cardiol 167(5):2149–2155 10.1016/j.ijcard.2012.05.099 22704868

[22] Je HG, Kim BH, Cho KI (2015). Correlation between patient-reported symptoms and ankle-brachial index after revascularization for peripheral arterial disease. Int J Mol Sci.

[23] Kalbaugh CA, Taylor SM, Blackhurst DW (2006). One-year prospective quality-of-life outcomes in patients treated with angioplasty for symptomatic peripheral arterial disease. J Vasc Surg.

[24] Keeling AN, Naughton PA, O’connell A (2008). Does percutaneous transluminal angioplasty improve quality of life?. J Vasc Interv Radiol.

[25] Lane R, Ellis B, Watson L (2014). Exercise for intermittent claudication. Cochrane Database Syst Rev.

[26] Lee JH, Cho KI, Spertus J (2012). Cross-cultural adaptation and validation of the peripheral artery questionnaire: Korean version for patients with peripheral vascular diseases. Vasc Med.

[27] Leicht AS, Crowther RG, Muller R (2011). The effects of including quality of life responses in models to predict walking performance of patients with intermittent claudication. Eur J Vasc Endovasc Surg.

[28] Maksimovic M, Vlajinac H, Marinkovic J (2014). Health-related quality of life among patients with peripheral arterial disease. Angiology.

[29] Malagoni AM, Vagnoni E, Felisatti M (2011). Evaluation of patient compliance, quality of life impact and cost-effectiveness of a “test in-train out” exercise-based rehabilitation program for patients with intermittent claudication. Circ. J..

[30] Mangiafico RA, Messina R, Attina T (2000). Impact of a 4-week treatment with prostaglandin E1 on health-related quality of life of patients with intermittent claudication. Angiology.

[31] Mehta T, Venkata Subramaniam A, Chetter I (2003). Disease-specific quality of life assessment in intermittent claudication: review. Eur J Vasc Endovasc Surg.

[32] Morgan MBF, Crayford T, Murrin B (2001). Developing the vascular quality of life questionnaire: a new disease-specific quality of life measure for use in lower limb ischemia. J. Vasc. Surg..

[33] Nicolai SP, Kruidenier LM, Rouwet EV (2009). The walking impairment questionnaire: an effective tool to assess the effect of treatment in patients with intermittent claudication. J Vasc Surg.

[34] Oka RK, Sanders MG (2005). The impact of type 2 diabetes and peripheral arterial disease on quality of life. J Vasc Nurs.

[35] Pell JP (1995). Impact of intermittent claudication on quality of life. The Scottish Vascular Audit Group. Eur J Vasc Endovasc Surg.

[36] Prevost A, Lafitte M, Pucheu Y (2015). Education and home based training for intermittent claudication: functional effects and quality of life. Eur J Prev Cardiol.

[37] Shuriquie MA, Banikhaled MH, Shudifat RM (2016). Perception of quality of life among patients with peripheral arterial disease in Jordan. Jordan Med J.

[38] Spertus J, Jones P, Poler S (2004). The peripheral artery questionnaire: a new disease-specific health status measure for patients with peripheral arterial disease. Am. Heart J..

[39] Tsai JC, Chan P, Wang CH (2002). The effects of exercise training on walking function and perception of health status in elderly patients with peripheral arterial occlusive disease. J. Intern. Med..

[40] Virkkunen J, Venermo M, Saarinen J (2008). Impact of endovascular treatment on clinical status and health-related quality of life. Scand J Surg.

[41] Ware JE, Sherbourne CD (1992). The MOS 36-item short-form health survey (SF-36): I. conceptual framework and item selection. Med Care.

[42] Yan BP, Lau JY, Yu CM (2011). Chinese translation and validation of the Walking Impairment Questionnaire in patients with peripheral artery disease. Vasc Med.

